# Response of *Sphagnum* Peatland Testate Amoebae to a 1-Year Transplantation Experiment Along an Artificial Hydrological Gradient

**DOI:** 10.1007/s00248-014-0367-8

**Published:** 2014-02-01

**Authors:** Katarzyna Marcisz, Bertrand Fournier, Daniel Gilbert, Mariusz Lamentowicz, Edward A. D. Mitchell

**Affiliations:** 1 Department of Biogeography and Palaeoecology, Adam Mickiewicz University in Poznań, Dzięgielowa 27, 61-680 Poznań, Poland; 2 Laboratory of Wetland Ecology and Monitoring, Adam Mickiewicz University in Poznań, Dzięgielowa 27, 61-680 Poznań, Poland; 3 Laboratory of Soil Biology, University of Neuchâtel, Rue Emile-Argand 11, 2000 Neuchâtel, Switzerland; 4 Laboratoire de Chrono-environment, UMR 6249 CNRS, Université de Franche-Comté, 16 route de Gray, 25030 Besancon Cedex, France

## Abstract

Peatland testate amoebae (TA) are well-established bioindicators for depth to water table (DWT), but effects of hydrological changes on TA communities have never been tested experimentally. We tested this in a field experiment by placing *Sphagnum* carpets (15 cm diameter) collected in hummock, lawn and pool microsites (*origin*) at three *local conditions* (dry, moist and wet) using trenches dug in a peatland. One series of samples was *seeded* with microorganism extract from all microsites. TA community were analysed at T0: 8–2008, T1: 5–2009 and T2: 8–2009. We analysed the data using conditional inference trees, principal response curves (PRC) and DWT inferred from TA communities using a transfer function used for paleoecological reconstruction. Density declined from T0 to T1 and then increased sharply by T2. Species richness, Simpson diversity and Simpson evenness were lower at T2 than at T0 and T1. Seeded communities had higher species richness in pool samples at T0. Pool samples tended to have higher density, lower species richness, Simpson diversity and Simpson Evenness than hummock and/or lawn samples until T1. In the PRC, the effect of *origin* was significant at T0 and T1, but the effect faded away by T2. *Seeding* effect was strongest at T1 and lowest vanished by T2. *Local condition* effect was strong but not in line with the wetness gradient at T1 but started to reflect it by T2. Likewise, TA-inferred DWT started to match the experimental conditions by T2, but more so in hummock and lawn samples than in pool samples. This study confirmed that TA responds to hydrological changes over a 1-year period. However, sensitivity of TA to hydrological fluctuations, and thus the accuracy of inferred DWT changes, was habitat specific, pool TA communities being least responsive to environmental changes. Lawns and hummocks may be thus better suited than pools for paleoecological reconstructions. This, however, contrasts with the higher prediction error and species’ tolerance for DWT with increasing dryness observed in transfer function models.

**Electronic supplementary material:**

The online version of this article (doi:10.1007/s00248-014-0367-8) contains supplementary material, which is available to authorized users.

## Introduction

Peatlands are key ecosystems in the global carbon cycle. Covering 3 % of world’s land area, they contain 30 % of all global soil carbon [1]. Northern Hemisphere *Sphagnum*-dominated peatlands cover vast surfaces of North America and Eurasia. They are nevertheless among the most vulnerable ecosystems to global warming [2]. Drought [3] can also potentially feedback positively to warming by causing losses of C as CO_2_ and CH_4_ [4, 5]. Understanding peatland response to ongoing climate change is therefore crucial for improving global climate projections. Moreover, by providing archives of past environmental and specially hydrological changes [6], peatlands represent key ecosystems to understand the long-term impact of climate change.

Testate amoebae (TA) are common protozoa in *Sphagnum* peatlands. As top predators within microbial food webs, they have a direct impact on the functioning of theses ecosystems [7]. Moreover, TA produce decay-resistant shells that are commonly used to reconstruct past peatland hydrology (usually water table depth) [8–10], as well as trophic status [11]. These paleo-reconstructions include periods such as the medieval climate anomaly analogous to the current increase in extreme events [12] providing essential information about ecosystems’ response to global change. The use of TA in palaeoecology as well as biomonitoring [13–15] is, however, based solely on descriptive studies of current patterns of community composition along hydrological gradients from which transfer functions are developed and subsequently used for palaeoecological reconstructions.

Only few experimental studies have so far been done on TA. Lousier manipulated soil moisture in the Rocky Mountain aspen woodland and showed that increased soil moisture is significantly correlated with the total number of living TA individuals [16, 17]. Beyens and co-workers [18] assessed the response of testate amoebae to soil temperature and precipitation manipulations. Long-term field warming and precipitation manipulation experiments under sub-arctic conditions revealed stronger warming effects in the growing season than in winter [19, 20]. TA abundance and community structure were also shown to respond to peatland drainage [21, 22]. However, experimental approaches investigating the response of testate amoebae to changes in hydrological conditions are lacking.

The aim of the study was to assess the response of TA communities to changes in water table depth in a transplantation experiment simulating hydrological changes. Moss patches collected in hummocks, lawns and pools (origin) were transplanted to wet, moist or dry position in a semi-controlled setting in a peatland in the French Jura Mountains. Pooled microbial communities extracted from the three original habitats (i.e. hummocks, lawns and pools) were added to a second set of samples to provide the full potential community. We hypothesised that (1) transplantation effects on TA community structure would be strongest in the most contrasted situations (i.e. transplanting mosses from pool to dry position and the other way around) and (2) the effect of the origin would fade away with time as the TA communities adapt to the experimental position; in parallel to this the DWT inferred from communities would initially match the origin position and would then gradually match the experimental position over time. With respect to seeding with mixed communities the two alternative hypotheses were that DWT inferred from seeded communities could change either faster or slower than non-seeded communities to match the conditions of the experimental position. A faster response would be predicted based on the fact that seeding provided dry as well as wet indicators. If species poorly adapted to the new conditions die rapidly while the better-adapted ones develop faster, inferred DWT would more rapidly match the experimental conditions. Alternatively, according to the insurance hypothesis [23, 24], adding mixed communities may provide a much more diverse (taxonomically and functionally) community, which would adapt less rapidly to changing conditions.

## Methods

### Study Site

**Fig. 1:**
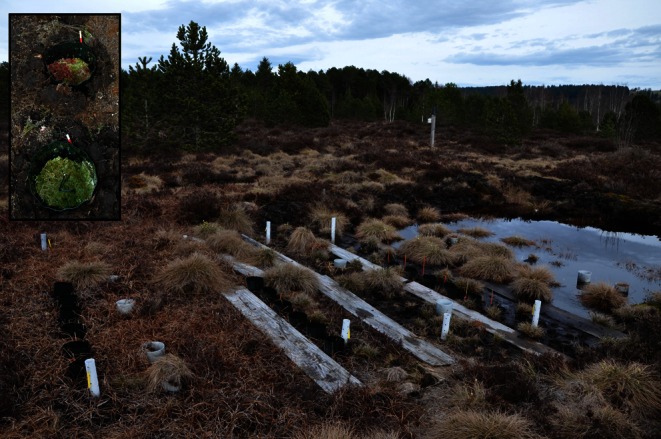
Study site in Le Russey Bog, French Jura. The picture shows one of the three experimental trenches. Green collars contain the transplanted *Sphagnum* patches (shown in the *inset*) placed along lines corresponding to dry, moist and wet situation

Le Russey is a cutover oligotrophic bog, located in the French Jura Mountains at 820 m a.s.l. (coordinates: 47°10′43.65″ N; 6°47′24.71″ E, Fig. 1). The local climate is characterised by mountain, oceanic and continental influences [25]. The vegetation is dominated by *Sphagnum capillifolium*, *Sphagnum fallax*, *Carex rostrata*, *Vaccinium uliginosum*, *Vaccinium oxycoccos*, *Eriophorum angustifolium*, *Eriophorum vaginatum*, *Calluna vulgaris* and *Polytrichum strictum*. This site was intensively exploited for peat extraction between 1968 and 1984. Currently the bog covers approximately 27 ha [25]. The study was conducted in the western, formerly exploited, part of the peatland, where three trenches were dug out as a part of a former experiment [26].

### Field Experiment

The experiment was conducted on three trenches (A–C; Fig. 1), which were dug out to create a gradient of hydrological conditions used in a former study [26]. In each trench, poly(vinyl chloride) collars about 15 cm diameter and 10 cm in length were placed at the surface of the trenches along three lines (referred to as “position”: dry, moist and wet), six collars on each line.

*Sphagnum* plugs of the exact same size as the collars were collected on the same peatland from three habitats referred to as “origin”: pools (*S. fallax*; origin P), lawns (*S. fallax*; origin L) and hummocks (*S. capillifolium*; origin H), and placed in plastic collars in the trenches (two per origin per line, with randomised positions) (Fig. 1, inset). Pools were composed of *S. fallax* and *C. rostrata*. Lawns contained *S. fallax, S. capillifolium* (isolated individuals), *V. oxycoccos*, *C. rostrata*, and *E. vaginatum*. Hummocks were dominated by *S. capillifolium*, *Polytrichum strictum*, *Calluna vulgaris* and *V. uliginosum*. The average depth to water table was 34.4, 16.0 and 6.0 cm in the hummock, lawn and pool habitats, respectively, on the day the *Sphagnum* plugs were harvested. Two sets of collars were set up. One of these was “seeded” with pooled extract from the three same habitats in order to provide the full potential community from the onset of the experiment. This extract was obtained by washing hummock, lawn and pool *Sphagnum* in bog water in a large bucket. A 250-ml volume of this extract was slowly poured over each patch. The experiment thus consisted of 54 different *Sphagnum* plots (3 *origins ×* 3 *local conditions ×* 2 initial community manipulation (*seeding*) *×* 3 replicates) (experimental trenches, see Supplementary Table 1 for details).

Moss samples were collected from the collars with transplanted *Sphagnum* three times over a 1-year period: in August 2008 (T0), May 2009 (T1) and August 2009 (T2). *Sphagnum* samples were placed in 4 % glutaraldehyde and stored at 4 °C in the dark to allow separating living and encysted from dead testate amoebae [27].

Depth to water table (DWT) was recorded at each sampling time in piezometers inserted at both ends of each line to determine the actual DWT influencing the communities in the moss carpets. As the height of the moss carpets changed over time due to moss growth and decomposition, we recorded the difference between the top of the moss carpet and the peat to obtain an individual DWT value for each plot. The average DWT in the experimental plots over the three sampling periods was 31.8 cm in the dry position, 19.5 in the moist position and 9.0 in the wet position, thus approximately matching the contrasts of the habitats of origin.

### Laboratory Analyses

Testate amoebae were extracted from *Sphagnum* following the method described by Booth et al. [28] and Jassey [29]. Coarse materials were removed using a 300-μm mesh size, and the filtrate analysed for TA. *Lycopodium* tablets were added to samples for density calculations [30]. Shells were counted at ×200 and ×400 magnification and identified at the highest taxonomic resolution using several keys and taxonomic monographs [31–35]. We recorded living (active + encysted) and dead amoebae separately, aiming for a total of 100 living individuals per sample. Only data on living individuals was used in the analyses. The >300 μm fraction of the *Sphagnum* samples were dried at 60 °C for 24 h and weighed to express density on a dry weight basis.

### Numerical Analyses

We first assessed the impact of the treatments on testate amoeba total density, species richness, Simpson diversity (N2) and Evenness (E2) (Hill). We described the changes of these indices (1) between seeded and non-seeded plots, (2) among origins, (3) among position and (4) over time. Given the broad temporal window between two sampling sessions and the relative short generation time (about 1 week of TA [36]), we considered here time as a factor and analysed it alongside treatment variables. Conditional Inference Trees (CIT)—a method that uses binary recursive partitioning [37]—were constructed for each index in order to characterise the responses of TA diversity indices to the treatments (origin, position, and community manipulations) and to temporal changes. CIT allow identifying which descriptive variables contribute most to data structure through permutation-based significance tests.

We then assessed the impact over time of origin, community manipulation (*seeding*) and position (*local condition*) on the structure of TA communities (percentage data) using principal response curves (PRC), a variant of redundancy analyses specially designed for repeated observation designs [38]. TA percentage community data was Hellinger transformed prior to analyses [39]. PRCs were computed separately for the different treatments (origin, community and position). This allowed us to assess the direction and magnitude of responses of TA species to each treatment.

We hypothesized that the DWT inferred from TA communities should gradually fit the experimental conditions (*local conditions*) as the species adapt to the new situation. To test this, we calculated the DWT inferred from the TA communities using an existing transfer function from the Swiss Alps [40]. We chose the transfer function from the Swiss Alps rather than a more local one from the Jura Mountains [41] as the Alpine data set is larger. Model performance was assessed using root mean squared error of prediction, average bias, maximum bias, and the correlation between observed and predicted values as assessed by bootstrap cross-validation [42]. For the comparison of the best performing, weighted averaging model (WA) was used. DWT was inferred from TA communities of different *origins* (hummock, lawn and pool) placed at different positions (D, dry; M, moist; W, wet) sampled at T0 (August 2008), T1 (May 2009) and T2 (August 2009), and either seeded or not.

All analyses were done using R [43] and the packages “vegan” [44] and “party” [37], except for the transfer function calculations that were performed with the software C2 [45].

## Results

### Testate Amoeba Community Composition

We recorded 40 living testate amoeba taxa in the 162 samples (Supplementary Table 2). Overall, *Archerella flavum* and *Hyalosphenia papilio* dominated the community and together accounted for 58.4 % of the total TA community. *Assulina seminulum*, *Assulina muscorum*, *Nebela tincta*, *Nebela militaris*, *Corythion dubium*, *Euglypha compressa*, *Euglypha tuberculata*, and *Hylosphenia elegans* each contributed on average to >1 % and together accounted for an additional 34.1 % of the community. Overall, *A. flavum* and *H. papilio* were co-dominant at T0 in seeded and non-seeded plots, but seeding reduced the contribution of *A. flavum* to the community (seeded: *A. flavum =* 19.5 % and *H. papilio* = 34.2 %, non-seeded: 30 and 29.2 %, respectively). By T1 seeding effect on *A. flavum* reverted: its relative abundance declined to 15 % in non-seeded communities but increased slightly in seeded plots (22.9 %) while *H. papilio* remained dominant in both (32.9 and 29.2 % in seeded and non-seeded, respectively). By T2 communities shifted to a strong dominance of *A. flavum* (63.2 and 64.3 % in seeded and non-seeded, respectively), while *H. papilio* decreased sharply (5 and 4.1 %).

### Testate Amoeba Density and Taxonomic Diversities

**Fig. 2:**
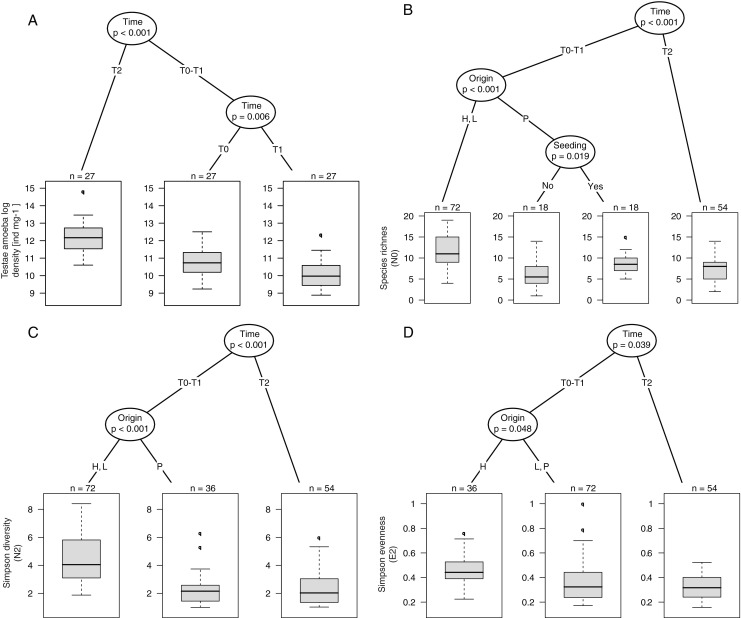
Conditional inference trees assessing the impact of community manipulations (seeded vs. non-seeded; *Seeding*), transplantations from different habitats (hummocks, lawns and pools; *Origin*), to different local hydric conditions (dry, moist, wet; *Local conditions*) and seasonal changes (T0, August 2008; T1, May 2009; and T2, August 2009) on testate amoeba density and diversity (i.e. species richness, Simpson diversity and evenness) in the experimental plots of Le Russey bog, French Jura. *p* values are given below variable names. *Boxplots* show the distribution of response variables per groups; *n* indicates the number of observations within each group

Total testate amoeba density ranged from 7.16 to 71.2 individuals per milligram dry weight of *Sphagnum* (Supplementary Table 3). Density was significantly higher at T2 than at T0 and T1 and decreased between T0 and T1, regardless of community manipulation, origin or position (Fig. 2a). Species richness varied in relation to time with significant effects of origin and seeding at T0 and T1 (Fig. 2b). At T0 and T1 species richness was higher in samples from hummocks and lawns. In samples transplanted from pools, seeding resulted in a significant increase in species richness. Simpson diversity showed a similar pattern to species richness (Fig. 2c). However, seeding did not result in a significant increase in pools at T0. Simpson evenness also differed in relation to time and origin being higher at T0 and T1 than at T2 (Fig. 2d). At T0 and T1, samples originating from hummocks had significantly higher evenness values than samples from pools and lawns where the mixotrophic species *H. papilio* and *A. flavum* strongly dominated the community.

### Responses of Community Composition to Treatments and Inferred DWT Over Time

**Fig. 3:**
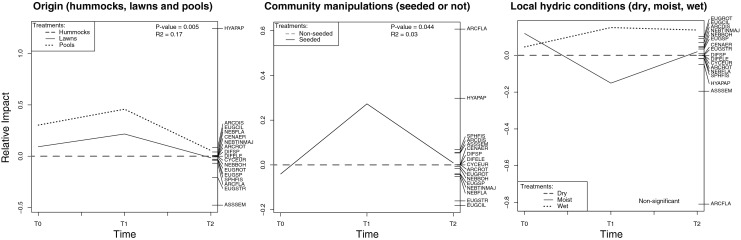
Principal response curves assessing the impact over time of habitat of origin (hummocks, lawns and pools; *Origin*), community manipulations (seeded vs. non-seeded; *Seeding*), local hydric conditions (dry, moist, wet; *Local conditions*) on testate amoeba community structure in the experimental plots of Le Russey bog, French Jura. Species scores are displayed on the *right vertical axis*

The three PRC models (Fig. 3) showed that origin and, to a lesser extent, community manipulations significantly impacted testate amoeba community composition (respectively 17 and 3 % of variance explained, *p* = 0.005 and 0.044). Contrary to expectation, both effects peaked at T1 rather than T0. However, in line with our hypothesis, the effects of community manipulation (seeding/non-seeding) and transplantation disappeared at T2. Although the communities in the wet position diverged from those in the other two situations from T1 onwards, the overall PRC model for *local condition* was not significant. However, individual RDA with measured DWT, *origin* and *seeding* as explanatory variables showed that the effect of *local condition* on the communities became significant by T2 (adjusted *R* ^2^ = 8.4 %, *p* value for variable “DWT” = 0.002; Supplementary Table 4).

**Fig. 4:**
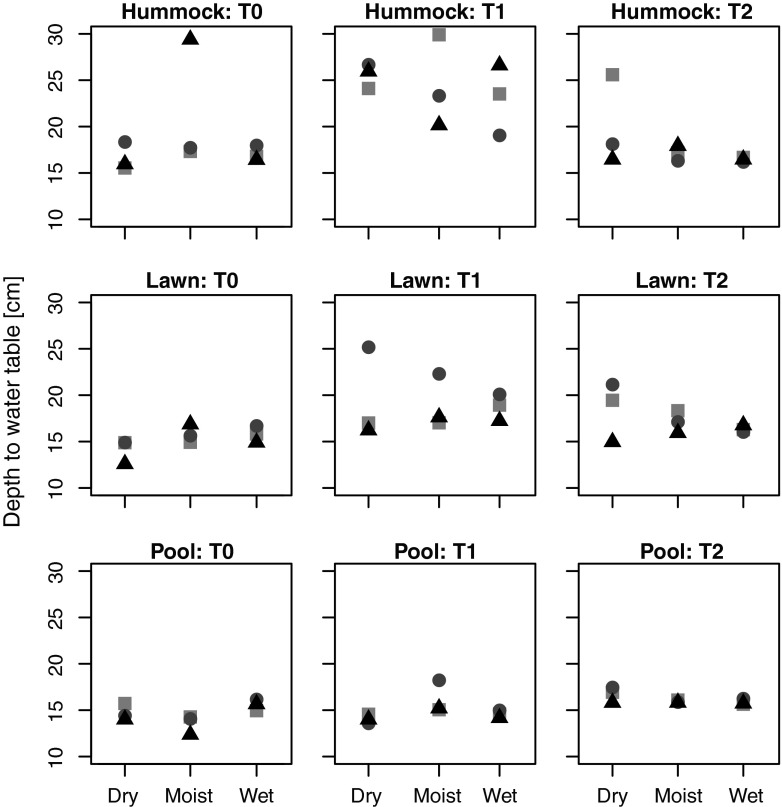
Depth to water table (DWT, in cm) inferred (using the transfer function model from Engadine [40]) from testate amoeba communities of different *origins* (hummock, lawn and pool) placed in different *local conditions* (*D* dry, *M* moist, *W* wet) and sampled at T0 (August 2008), T1 (May 2009) and T2 (August 2009) in the experimental plots of Le Russey bog, French Jura. Average measured DWT was 31.8, 19.5 and 9.0 cm in the dry, moist and wet positions, respectively. Data from non-seeded communities (see Supplementary Figure 4 for corresponding figure showing seeded community data). The *symbols* represent the different replicates

Inferred DWT values differed strongly from values measured in the experimental trenches at T0 and increasingly fitted the local conditions over time for all transplanted samples, in the seeded as well as non-seeded plots (Fig. 4, Supplementary Fig. 4). Inferred DWT increased between T0 and T1 and decreased between T1 and T2. These trends were clearest in samples collected in hummocks, relatively marked in samples from lawns and almost non-existent in pool samples, and were more marked in non-seeded than in seeded plots. Inferred DWT was on average lower (i.e. wetter) in seeded plots at T0 and T1, indicating that seeding favoured “wet indicators”, and the range of inferred DWT was lower in seeded plots (13.5–25.5 cm in non-seeded vs. 12.2–19.4 cm in seeded plots).

## Discussion

### Effects of Hydrological Changes

This is the first experimental study testing the effect of hydrological changes on testate amoeba communities in *Sphagnum* peatlands. Given the well-established relationship between TA communities and DWT in peatlands and other experimental evidence for rapid responses to moisture manipulation [16, 17], we expected to see a clear DWT effect on communities, which would also be reflected by clear shifts in TA-inferred DWT. We hypothesised that TA community structure would change most quickly and clearly in contrasted situations, that the effect of the origin would decline over time and that inferred DWT would gradually match that of the local conditions. Our results partly supported these hypotheses. Although TA community structure changed, these changes were not clearer in more contrasted transitions. An important result, however, is that hummock communities changed more than pool communities, suggesting that TA communities from dry microsites may be more sensitive to micro-environmental changes than pool communities, possibly because they contain a broader mix of dry and wet indicators than wetter microsites. This result could be seen as counter-intuitive given results from transfer-function models, which regularly show better predictive power at the wet end of the hydrological gradient while DWT beyond about 30 cm cannot be reliably predicted [41, 46]. However, we tested the effect of increased wetness on hummock communities and not the effect of extreme drought; it is therefore likely that an extreme drought experiment would not reveal clear TA community changes. A recent experimental study simulating increased rainfall magnitude, intensity and frequency on *Sphagnum fuscum*-dominated vegetation at Abisko (Northern Sweden) also yielded unexpected results [47]. Contrary to expectation the density of hygrophilous taxa such as *A. flavum* and *H. papilio* decreased, possibly due to changes or fluctuation in water chemistry (the simulated rain was achieved using stream water with a near-neutral pH as opposed to the likely very acidic pH in the *S. fuscum* mosses) and/or prey or food items.

### Effects of Origin and Seeding

Contrary to our expectation, the effect of origin remained significant until the end of the experiment. This suggests that the original community signature remained significant in our study despite the contrasted experimental water table positions imposed to the communities. This could suggest a tighter than expected relationship between TA communities and *Sphagnum* species (e.g. suggesting possibly chemical effects related to compounds released by the mosses) or the structure of the moss carpet (i.e. microclimatic effects overriding the DWT effect).

Seeding significantly affected community composition at T0 and even more so at T1, but this effect had disappeared by T2 (Figs. 3 and 4, Supplementary Fig. 4). This suggests that, although we had added a considerable amount of microorganisms in the seeded plots, this effect was not a lasting one. The effect of seeding brings partial support to both the hypothesis of a faster response of enhanced communities as well as the insurance hypothesis. Indeed in hummock and lawn samples seeding reduced the contrasts between dry and wet local position at T2, in agreement with the insurance hypothesis. However the opposite is true for pool samples. This suggests that pool communities lacked dry indicators at T0 and therefore that seeding allowed these communities to shift faster over time and to reflect the local hydrology.

### General Stress Response

Species richness, Simpson diversity and evenness decreased over the course of the experiment (Supplementary Figs. 1–3). This suggests that, within the timeframe of the experiment, the stress caused by the experiment (manipulation of the moss carpet and changes in DWT) could not be compensated by immigration of new species (potentially better adapted to the new conditions and absent from the original samples) and that the seeding effect did not last beyond T1 (i.e. May, end of winter—9 months after the beginning of the experiment). Our experimental setup might have caused too much stress on the moss and microbial communities to allow the expected changes to occur. Further experiments will be needed to better understand the precise mechanism influencing *Sphagnum* TA communities.

A somewhat striking and counter-intuitive pattern is the drier inferred conditions at T1 (i.e. higher DWT values), in non-seeded hummock plots. T1 corresponds to the spring sampling and thus reflects the treatment effects over the end of summer, autumn, winter and early spring. This counter-intuitive result may be explained by the capacity of hummock *Sphagnum* to retain relatively high moisture content thanks to the tight structure of the moss carpet and efficient capillarity. Under natural conditions, moisture conditions can indeed vary more in pools than in hummocks [48]. Thus although pools are generally wetter, in extreme dry periods they can dry out. By contrast *Sphagnum* species that build hummocks (e.g. *S. fuscum*, *S. capillifolium*) are able to maintain stable moist conditions [49] even when the water table is low. We would therefore have expected lawn and pool communities to be more strongly affected by lowered water table. However, our results rather show that hummock communities were more affected by the experimental manipulation than lawn and pool communities at T1. We interpret this as being due to the disruption of the tight hummock moss carpet structure. Indeed, although the size of the experimental plots were large by microbial standards (15 cm diameter and 10 cm high) and we sampled the plugs carefully to maintain the structure of the moss carpet, this size may not have been large enough to allow the mosses to maintain their moisture during dry periods. Indeed, we observed that the hummock moss carpets suffered from drought effects and their tight structure was lost over the course of the experiment. This result is in line with the lower density observed at T1 as compared to T0 and T2 (Fig. 2a). By T2, however, pool (seeded and non-seeded) and seeded lawn communities indicated significantly lower water table than at T0 but this was not the case for hummock communities and non-seeded lawn communities. This suggests that TA species indicative for wet conditions on average tolerated the experimental conditions less well than drier indicators. This interpretation therefore suggests that hummock *Sphagnum* mosses are less tolerant to disturbance and this in turn affects TA communities, while in the case of TA, lowest tolerance to disturbance is observed among pool species. These contradictory results would explain the lack of clear overall response to local hydrological conditions (Fig. 1c).

### Implications for Bioindication and Paleoecology

Testate amoeba analysis is now a well-established tool for palaeo-hydrological reconstructions [40, 50, 51]. However, beyond major differences among micro-habitats (e.g. pool-hummock gradient), the spatial and seasonal patterns of TA density, diversity and community structure and their response to micro-environmental changes remain poorly understood. Our results suggest that DWT changes may not affect TA communities in the same way in hummocks than in lawns or pools. This is an important issue in case of the past water table depth reconstructions and interpretation of palaeo-hydrological changes, as when describing data obtained from peat cores, it is essential to apply reliable testing sets based on detailed information about species-environment relations. Our results suggest that lawns and hummocks are better suited than hollows and pools for palaeohydrological reconstruction. This contrasts with the higher prediction error and species’ tolerance for DWT with increasing dryness observed in transfer function models. However, the specific experimental conditions (e.g., disruption of the moss carpet structure) might have affected TA communities. Further work is necessary to better interpret past changes in TA communities and thus to improve the accuracy of palaeo-environmental reconstruction. Such work is necessary to better interpret past changes in TA communities and thus to improve the accuracy of palaeo-environmental reconstruction.

## Electronic Supplementary Material

Below is the link to the electronic supplementary material.

Supplementary Table 1 Experimental design and measured depth to water table (DWT) at the time of setup and at each sampling time. (DOCX 23.8 kb)

Supplementary Table 2 Relative abundance of testate amoeba taxa observed in the experimental plots at Le Russey, French Jura in seeded and non-seeded plots over time. T0, August 2008; T1, May 2009 and T2, August 2009. (DOCX 24.5 kb)

Supplementary Table 3 Density [ind/mg] of testate amoeba taxa observed in the experimental plots at Le Russey, French Jura in seeded and non-seeded plots over time. (DOCX 26.4 kb)

Supplementary Table 4 Summary results of the RDA model on testate amoeba community data showing changes in significance of the origin (hummock, lawn and pool), seeding (adding pooled community extract from hummock, lawn and pool) and local condition (depth to water table measured in the experimental plots) over the course of the experiment (T0, August 2008; T1, May 2009 and T2, August 2009). Significant values are indicated in bold. (DOCX 11.9 kb)

Supplementary Figure 1 Changes over time in density [ind/mg], species richness (N0), Simpson diversity (N2) and evenness (E2 = N2/N0) of living testate amoebae of communities seeded vs. non-seeded with mixed extract from hummock, lawn and pool habitats, showing data of samples from hummock, lawn and pool habitats (*origin*) placed at high, intermediate and low water table position (*local condition*) in the experimental trenches of Le Russey bog, French Jura. T0, August 2008; T1, May 2009 and T2, August 2009. (JPEG 378 kb)

High Resolution Image (EPS 720 kb)

Supplementary Figure 2 Changes over time in density [ind/mg], species richness (N0), Simpson diversity (N2) and evenness (E2 = N2/N0) of living testate amoebae for samples collected in hummock, lawn and pool habitats, showing data of samples seeded and not seeded with mixed extract from the three habitats (origin) and placed at high, intermediate and low water table position (local condition) in the experimental trenches of Le Russey bog, French Jura. T0, August 2008; T1, May 2009 and T2, August 2009. (JPEG 397 kb)

High Resolution Image (EPS 725 kb)

Supplementary Figure 3 Changes over time in density [ind/mg], species richness (N0), Simpson diversity (N2) and evenness (E2 = N2/N0) of living testate amoebae for samples placed at high, intermediate and low water table position (local condition) the experimental trenches of Le Russey bog, French Jura, showing data of samples collected in hummock, lawn and pool habitats (origin) seeded and not seeded with mixed extract from the three habitats. T0, August 2008; T1, May 2009 and T2, August 2009. (JPEG 372 kb)

High Resolution Image (EPS 580 kb)

Supplementary Figure 4 Depth to water table (DWT, in cm) inferred (using the transfer function model from Engadine [40]), from testate amoeba communities sampled at T0, T1 and T2 in the plots of different origins (hummock, lawn and pool) placed at different position (*D* dry, *M* moist, *W* wet). Data from communities seeded with mixed extract from hummock, lawn and pool habitats. See Fig. 4 for corresponding figure on non-seeded communities. (JPEG 196 kb)

High Resolution Image (EPS 444 kb)
