# Supplementary material for: Response of Sphagnum Peatland Testate Amoebae to a 1-Year Transplantation Experiment Along an Artificial Hydrological Gradient
Source: Microb Ecol. 2014 Feb 1;67(4):810–8. doi: 10.1007/s00248-014-0367-8 (PMC3984440; doi:10.1007/s00248-014-0367-8)
Supplement: Supplementary file 1 — Experimental design and measured depth to water table (DWT) at the time of setup and at each sampling time. (DOCX 23.8 kb) [file 248_2014_367_MOESM1_ESM.docx]

| **Supplementary table 1.** Experimental design and measured depth to water table (DWT) at the time of setup and at each sampling time. | | | | | | | | | |
| --- | --- | --- | --- | --- | --- | --- | --- | --- | --- |
|  |  |  |  |  |  | DWT [cm] | | | |
| Plot Code | Trench | Local condition ^1^ | Origin ^2^ | Community ^3^ |  | T0 origin | T0-Trench | T1 | T2 |
| A-D-L+ | A | D | L | + |  | 16 | 28,2 | 27,2 | 27,2 |
| A-D-P- | A | D | P | - |  | 6 | 28,2 | 26,7 | 26,7 |
| A-D-H+ | A | D | H | + |  | 30 | 28,2 | 27,2 | 27,2 |
| A-D-L- | A | D | L | - |  | 16 | 28,2 | 29,2 | 29,2 |
| A-D-H- | A | D | H | - |  | 37 | 28,2 | 28,2 | 28,2 |
| A-D-P+ | A | D | P | + |  | 6 | 28,2 | 27,2 | 27,2 |
| A-M-L+ | A | M | L | + |  | 16 | 20,5 | 18,5 | 18,5 |
| A-M-L- | A | M | L | - |  | 16 | 20,5 | 19,5 | 19,5 |
| A-M-H+ | A | M | H | + |  | 31 | 20,5 | 17,5 | 17,5 |
| A-M-P- | A | M | P | - |  | 6 | 20,5 | 19,0 | 19,0 |
| A-M-H- | A | M | H | - |  | 28 | 20,5 | 20,5 | 20,5 |
| A-M-P+ | A | M | P | + |  | 6 | 20,5 | 18,0 | 18,0 |
| A-W-P- | A | W | P | - |  | 6 | 6,7 | 12,7 | 12,7 |
| A-W-L+ | A | W | L | + |  | 16 | 6,7 | 5,2 | 5,2 |
| A-W-L- | A | W | L | - |  | 16 | 6,7 | 5,2 | 5,2 |
| A-W-H- | A | W | H | - |  | 30 | 6,7 | 9,7 | 9,7 |
| A-W-P+ | A | W | P | + |  | 6 | 6,7 | 6,7 | 6,7 |
| A-W-H+ | A | W | H | + |  | 38 | 6,7 | 3,7 | 3,7 |
| B-D-H- | B | D | H | - |  | 43 | 31,9 | 29,9 | 29,9 |
| B-D-H+ | B | D | H | + |  | 35 | 31,9 | 30,9 | 30,9 |
| B-D-L- | B | D | L | - |  | 16 | 31,9 | 29,9 | 29,9 |
| B-D-P- | B | D | P | - |  | 6 | 31,9 | 29,9 | 29,9 |
| B-D-P+ | B | D | P | + |  | 6 | 31,9 | 29,9 | 29,9 |
| B-D-L+ | B | D | L | + |  | 16 | 31,9 | 29,9 | 29,9 |
| B-M-H- | B | M | H | - |  | 30 | 21,4 | 19,0 | 19,0 |
| B-M-L+ | B | M | L | + |  | 16 | 21,4 | 18,4 | 18,4 |
| B-M-H+ | B | M | H | + |  | 30 | 21,4 | 19,4 | 19,4 |
| B-M-P- | B | M | P | - |  | 6 | 21,4 | 19,4 | 19,4 |
| B-M-P+ | B | M | P | + |  | 6 | 21,4 | 17,4 | 17,4 |
| B-M-L- | B | M | L | - |  | 16 | 21,4 | 18,4 | 18,4 |
| B-W-L+ | B | W | L | + |  | 16 | 12,1 | 12,1 | 12,1 |
| B-W-H+ | B | W | H | + |  | 35 | 12,1 | 12,1 | 12,1 |
| B-W-L- | B | W | L | - |  | 16 | 12,1 | 9,1 | 9,1 |
| B-W-H- | B | W | H | - |  | 40 | 12,1 | 12,1 | 12,1 |
| B-W-P- | B | W | P | - |  | 6 | 12,1 | 15,1 | 15,1 |
| B-W-P+ | B | W | P | + |  | 6 | 12,1 | 12,6 | 12,6 |
| C-D-P- | C | D | P | - |  | 6 | 37,9 | 36,4 | 36,4 |
| C-D-H- | C | D | H | - |  | 32 | 37,9 | 37,9 | 37,9 |
| C-D-P+ | C | D | P | + |  | 6 | 37,9 | 34,9 | 34,9 |
| C-D-L+ | C | D | L | + |  | 16 | 37,9 | 36,2 | 36,2 |
| C-D-L- | C | D | L | - |  | 16 | 37,9 | 35,9 | 35,9 |
| C-D-H+ | C | D | H | + |  | 41 | 37,9 | 35,9 | 35,9 |
| C-M-H- | C | M | H | - |  | 43 | 21,3 | 18,3 | 18,3 |
| C-M-P+ | C | M | P | + |  | 6 | 21,3 | 18,8 | 18,8 |
| C-M-L- | C | M | L | - |  | 16 | 21,3 | 18,3 | 18,3 |
| C-M-P- | C | M | P | - |  | 6 | 21,3 | 18,3 | 18,3 |
| C-M-H+ | C | M | H | + |  | 37 | 21,3 | 20,3 | 20,3 |
| C-M-L+ | C | M | L | + |  | 16 | 21,3 | 19,3 | 19,3 |
| C-W-H- | C | W | H | - |  | 30 | 8,9 | 7,4 | 7,4 |
| C-W-P- | C | W | P | - |  | 6 | 8,9 | 11,9 | 11,9 |
| C-W-L- | C | W | L | - |  | 16 | 8,9 | 6,9 | 6,9 |
| C-W-H+ | C | W | H | + |  | 30 | 8,9 | 7,9 | 7,9 |
| C-W-L+ | C | W | L | + |  | 16 | 8,9 | 4,9 | 4,9 |
| C-W-P+ | C | W | P | + |  | 6 | 8,9 | 5,4 | 5,4 |
|  |  |  |  |  |  |  |  |  |  |
|  |  |  |  |  |  | DWT [cm] | | | |
| Average DWT per origin and per local condition | | | |  |  | Origin | Local condition (T0-T2) | | |
| ***Origin*** |  |  |  |  |  |  |  |  |  |
| Hummock |  |  |  |  |  | 34,4 |  |  |  |
| Lawn |  |  |  |  |  | 16 |  |  |  |
| Pool |  |  |  |  |  | 6 |  |  |  |
|  |  |  |  |  |  |  |  |  |  |
| ***Local condition (T0-T2)*** | |  |  |  |  |  |  |  |  |
| Dry |  |  |  |  |  |  | 31,8 | | |
| Moist |  |  |  |  |  |  | 19,5 | | |
| Wet |  |  |  |  |  |  | 9,0 | | |
| 1) D: dry (highest position on trench), M: moist (intermediate position), W: wet (lowest position) | | | | | | | |  |  |
| 2) H: hummock, L: lawn, P: pool | | |  |  |  |  |  |  |  |
| 3) +: added community at T0 (mixed extract of hummock, lawn and pool), -: no initial manipulation of community | | | | | | | | |  |
