# Supplementary material for: Response of Sphagnum Peatland Testate Amoebae to a 1-Year Transplantation Experiment Along an Artificial Hydrological Gradient
Source: Microb Ecol. 2014 Feb 1;67(4):810–8. doi: 10.1007/s00248-014-0367-8 (PMC3984440; doi:10.1007/s00248-014-0367-8)
Supplement: Supplementary file 2 — Relative abundance of testate amoeba taxa observed in the experimental plots at Le Russey, French Jura in seeded and non-seeded plots over time. T0, August 2008; T1, May 2009 and T2, August 2009. (DOCX 24.5 kb) [file 248_2014_367_MOESM2_ESM.docx]

| **Supplementary Table 2.** Relative abundance of testate amoeba taxa observed in the experimental plots at Le Russey, French Jura in seeded and non-seeded plots over time. T0=August 2008, T1=May 2009 T2=August 2009. | | | | | | | | | | | | | | | | |
| --- | --- | --- | --- | --- | --- | --- | --- | --- | --- | --- | --- | --- | --- | --- | --- | --- |
|  |  |  |  |  |  |  |  |  |  |  |  |  |  |  |  |  |
|  |  | Seeded | | | | Non-seeded | | | | Overall | | | | Seeding effect (raw % change) | | |
| Taxon | Code | T0 | T1 | T2 | Average | T0 | T1 | T2 | Average | T0 | T1 | T2 | Average | T0 | T1 | T2 |
| Archerella flavum | ARCFLA | 19,5 | 22,9 | 63,2 | 35,2 | 30,0 | 15,0 | 64,3 | 36,4 | 24,7 | 19,0 | 63,7 | 35,7 | -10,5 | 7,9 | -1,1 |
| Hyalpsphenia papilio | HYAPAP | 34,2 | 32,9 | 5,0 | 24,0 | 29,2 | 29,2 | 4,1 | 20,8 | 31,7 | 31,1 | 4,5 | 22,7 | 5,0 | 3,7 | 0,9 |
| Assulina seminulum | ASSSEM | 7,1 | 8,1 | 11,5 | 8,9 | 6,4 | 11,2 | 10,9 | 9,5 | 6,8 | 9,6 | 11,2 | 9,16 | 0,8 | -3,2 | 0,6 |
| Nebela tincta | NEB TIN | 6,2 | 11,5 | 3,5 | 7,1 | 3,8 | 15,0 | 4,2 | 7,7 | 5,0 | 13,3 | 3,8 | 7,32 | 2,5 | -3,6 | -0,7 |
| Corythion dubium | CORDUB | 4,1 | 6,1 | 9,5 | 6,6 | 1,5 | 3,7 | 9,3 | 4,8 | 2,8 | 4,9 | 9,4 | 5,83 | 2,6 | 2,4 | 0,2 |
| Assulina muscorum | ASSMUS | 7,2 | 3,3 | 1,7 | 4,1 | 4,2 | 10,3 | 1,3 | 5,2 | 5,7 | 6,8 | 1,5 | 4,58 | 3,0 | -6,9 | 0,5 |
| Euglypha compressa | EUGCOM | 2,2 | 4,5 | 0,3 | 2,3 | 2,7 | 2,0 | 0,4 | 1,7 | 2,5 | 3,2 | 0,3 | 2,05 | -0,5 | 2,5 | -0,2 |
| Nebela militaris | NEBMIL | 2,5 | 2,7 | 0,3 | 1,9 | 3,7 | 1,5 | 0,8 | 2,0 | 3,1 | 2,1 | 0,5 | 1,91 | -1,2 | 1,2 | -0,4 |
| Hyalosphenia elegans | HYAELE | 4,6 | 2,1 | 0,2 | 2,3 | 1,9 | 1,7 | 0,1 | 1,2 | 3,3 | 1,9 | 0,2 | 1,86 | 2,7 | 0,4 | 0,1 |
| Euglypha tuberculata | EUGTUB | 2,7 | 0,8 | 0,7 | 1,4 | 1,6 | 1,0 | 1,8 | 1,4 | 2,2 | 0,9 | 1,2 | 1,43 | 1,2 | -0,2 | -1,0 |
| Euglypha ciliata | EUGCIL | 1,5 | 0,6 | 0,1 | 0,7 | 1,7 | 2,3 | 0,1 | 1,4 | 1,6 | 1,5 | 0,1 | 1,02 | -0,2 | -1,7 | 0,0 |
| Euglypha strigosa | EUGSTR | 0,8 | 0,0 | 0,2 | 0,3 | 2,1 | 1,4 | 0,2 | 1,2 | 1,5 | 0,7 | 0,2 | 0,71 | -1,4 | -1,4 | 0,0 |
| Arcella discoides | ARCDIS | 2,3 | 0,3 | 0,4 | 1,0 | 0,6 | 0,0 | 0,5 | 0,4 | 1,4 | 0,1 | 0,5 | 0,72 | 1,7 | 0,3 | -0,1 |
| Heleopera sphagni | HELSPH | 0,9 | 0,1 | 0,1 | 0,4 | 2,0 | 0,3 | 0,5 | 0,9 | 1,4 | 0,2 | 0,3 | 0,61 | -1,1 | -0,2 | -0,4 |
| Euglypha laevis | EUGLAE | 0,0 | 1,0 | 0,8 | 0,6 | 0,1 | 0,7 | 0,2 | 0,3 | 0,1 | 0,9 | 0,5 | 0,49 | 0,0 | 0,3 | 0,6 |
| Assulina scandinavica | ASSSCA | 0,3 | 0,5 | 0,4 | 0,4 | 0,5 | 0,6 | 0,3 | 0,5 | 0,4 | 0,6 | 0,4 | 0,44 | -0,2 | 0,0 | 0,0 |
| Euglypha rotunda | EUGROT | 0,7 | 0,3 | 0,0 | 0,3 | 1,0 | 0,4 | 0,3 | 0,5 | 0,8 | 0,3 | 0,1 | 0,42 | -0,3 | -0,1 | -0,2 |
| Centropyxis aculeata | CENACU | 0,8 | 0,2 | 0,0 | 0,3 | 0,8 | 0,7 | 0,1 | 0,5 | 0,8 | 0,5 | 0,0 | 0,41 | -0,1 | -0,5 | -0,1 |
| Nebela tincta major | NEBTINMAJ | 0,0 | 0,0 | 0,0 | 0,0 | 2,2 | 0,3 | 0,0 | 0,8 | 1,1 | 0,1 | 0,0 | 0,35 | -2,2 | -0,3 | 0,0 |
| Nebela bohemica | NEBBOH | 0,4 | 0,0 | 0,0 | 0,1 | 1,6 | 0,1 | 0,0 | 0,6 | 1,0 | 0,1 | 0,0 | 0,32 | -1,2 | -0,1 | 0,0 |
| Corythion pulchellum | CORPUL | 0,2 | 0,1 | 0,9 | 0,4 | 0,0 | 0,3 | 0,1 | 0,2 | 0,1 | 0,2 | 0,5 | 0,30 | 0,1 | -0,2 | 0,8 |
| Trinema enchelys | TRIENC | 0,2 | 0,6 | 0,2 | 0,3 | 0,3 | 0,2 | 0,1 | 0,2 | 0,2 | 0,4 | 0,2 | 0,28 | -0,1 | 0,4 | 0,1 |
| Physochila griseola | PHYGRI | 0,4 | 0,3 | 0,3 | 0,3 | 0,2 | 0,3 | 0,1 | 0,2 | 0,3 | 0,3 | 0,2 | 0,27 | 0,2 | 0,0 | 0,2 |
| Heleopera rosea | HELROS | 0,3 | 0,4 | 0,0 | 0,3 | 0,4 | 0,1 | 0,1 | 0,2 | 0,4 | 0,3 | 0,1 | 0,23 | -0,1 | 0,2 | 0,0 |
| Euglypha cristata | EUGCRI | 0,4 | 0,1 | 0,2 | 0,3 | 0,1 | 0,1 | 0,2 | 0,1 | 0,3 | 0,1 | 0,2 | 0,21 | 0,3 | 0,0 | -0,1 |
| Cyclopyxis arcelloides | CYCARC | 0,0 | 0,0 | 0,1 | 0,0 | 0,8 | 0,1 | 0,0 | 0,3 | 0,4 | 0,1 | 0,0 | 0,14 | -0,8 | -0,1 | 0,1 |
| Phryganella acropodia | PHRACR | 0,0 | 0,0 | 0,0 | 0,0 | 0,0 | 1,0 | 0,0 | 0,3 | 0,0 | 0,5 | 0,0 | 0,14 | 0,0 | -1,0 | 0,0 |
| Bullinularia indica | BULIND | 0,0 | 0,0 | 0,0 | 0,0 | 0,4 | 0,1 | 0,0 | 0,2 | 0,2 | 0,0 | 0,0 | 0,07 | -0,4 | -0,1 | 0,0 |
| Nebela flabellulum | NEBFLA | 0,0 | 0,1 | 0,0 | 0,0 | 0,1 | 0,2 | 0,1 | 0,1 | 0,0 | 0,2 | 0,0 | 0,07 | -0,1 | -0,2 | -0,1 |
| Sphenoderia fissirostris | SPHFIS | 0,0 | 0,3 | 0,0 | 0,1 | 0,0 | 0,0 | 0,0 | 0,0 | 0,0 | 0,2 | 0,0 | 0,06 | 0,0 | 0,3 | 0,0 |
| Euglypha sp. | EUGSP | 0,1 | 0,0 | 0,0 | 0,0 | 0,0 | 0,1 | 0,0 | 0,0 | 0,1 | 0,1 | 0,0 | 0,05 | 0,1 | -0,1 | 0,0 |
| Trigonopyxis arcula | TRIARC | 0,1 | 0,0 | 0,1 | 0,0 | 0,1 | 0,0 | 0,0 | 0,0 | 0,1 | 0,0 | 0,0 | 0,04 | 0,0 | 0,0 | 0,1 |
| Heleopera sylvatica | HELSYL | 0,1 | 0,0 | 0,0 | 0,0 | 0,1 | 0,0 | 0,0 | 0,0 | 0,1 | 0,0 | 0,0 | 0,03 | -0,1 | 0,0 | 0,0 |
| Hyalosphenia subflava | HYASUB | 0,0 | 0,1 | 0,1 | 0,0 | 0,0 | 0,0 | 0,0 | 0,0 | 0,0 | 0,0 | 0,0 | 0,03 | 0,0 | 0,1 | 0,1 |
| Arcella catinus | ARCCAT | 0,0 | 0,1 | 0,0 | 0,0 | 0,0 | 0,0 | 0,0 | 0,0 | 0,0 | 0,0 | 0,0 | 0,01 | 0,0 | 0,1 | 0,0 |
| Cryptodifflugia oviformis | CRYOVI | 0,0 | 0,0 | 0,0 | 0,0 | 0,0 | 0,0 | 0,0 | 0,0 | 0,0 | 0,0 | 0,0 | 0,01 | 0,0 | 0,0 | 0,0 |
| Arcella vulgaris | ARCVUL | 0,1 | 0,0 | 0,0 | 0,0 | 0,0 | 0,0 | 0,0 | 0,0 | 0,0 | 0,0 | 0,0 | 0,01 | 0,1 | 0,0 | 0,0 |
| Difflugia pulex | DIFPUX | 0,0 | 0,0 | 0,0 | 0,0 | 0,0 | 0,1 | 0,0 | 0,0 | 0,0 | 0,0 | 0,0 | 0,01 | 0,0 | -0,1 | 0,0 |
| Centropyxis aerophila | CENAER | 0,0 | 0,0 | 0,0 | 0,0 | 0,0 | 0,0 | 0,0 | 0,0 | 0,0 | 0,0 | 0,0 | 0,01 | 0,0 | 0,0 | 0,0 |
| Nebela collaris | NEBCOL | 0,0 | 0,0 | 0,0 | 0,0 | 0,0 | 0,0 | 0,0 | 0,0 | 0,0 | 0,0 | 0,0 | 0,01 | 0,0 | 0,0 | 0,0 |
| The following taxa are only recorded as dead (empty) shells: Arcella hemispherica, A. rotunda, Cyclopyxis eurystoma, Difflugia lanceolata, D. elegans, D. globulosa, Heleopera petricola, Padaungiella tubulata (=Nebela tubulata), Phryganella acropodia, Pseudodifflugia gracilis, Trinema lineare. | | | | | | | | | | | | | | | | |
