# Supplementary material for: Response of Sphagnum Peatland Testate Amoebae to a 1-Year Transplantation Experiment Along an Artificial Hydrological Gradient
Source: Microb Ecol. 2014 Feb 1;67(4):810–8. doi: 10.1007/s00248-014-0367-8 (PMC3984440; doi:10.1007/s00248-014-0367-8)
Supplement: Supplementary file 3 — Density [ind/mg] of testate amoeba taxa observed in the experimental plots at Le Russey, French Jura in seeded and non-seeded plots over time. (DOCX 26.4 kb) [file 248_2014_367_MOESM3_ESM.docx]

| **Supplementary Table 3.** Density [ing. mg^-1^] of testate amoeba taxa observed in the experimental plots at Le Russey, French Jura in seeded and non-seeded plots over time. | | | | | | | | | | | | | | | | |
| --- | --- | --- | --- | --- | --- | --- | --- | --- | --- | --- | --- | --- | --- | --- | --- | --- |
|  |  |  |  |  |  |  |  |  |  |  |  |  |  |  |  |  |
|  |  | Seeded | | | | Non-seeded | | | | Overall | | | | Seeding effect (raw % change) | | |
| Taxon | Code | T0 | T1 | T2 | Average | T0 | T1 | T2 | Average | T0 | T1 | T2 | Average | T0 | T1 | T2 |
| Archerella flavum | ARCFLA | 679 | 2367 | 56261 | 19769,1 | 1233 | 912,3 | 60789 | 20978,2 | 1912 | 3279 | 1E+05 | 40747,4 | -554,2 | 1454,7 | -4527,9 |
| Hyalpsphenia papilio | HYAPAP | 9203 | 3415 | 2357 | 4991,79 | 2998 | 3954 | 2034 | 2995,5 | 12201 | 7369 | 4391 | 7987,3 | 6205,1 | -539,6 | 323,4 |
| Assulina seminulum | ASSSEM | 779,4 | 764,2 | 9281 | 3608,28 | 374,1 | 447 | 5528 | 2116,26 | 1154 | 1211 | 14809 | 5724,54 | 405,2 | 317,3 | 3753,6 |
| Nebela tincta | NEB TIN | 775,2 | 1093 | 1830 | 1232,43 | 243 | 718,2 | 1872 | 944,389 | 1018 | 1811 | 3702 | 2176,82 | 532,2 | 374,3 | -42,3 |
| Corythion dubium | CORDUB | 392,8 | 454,1 | 8800 | 3215,66 | 83,6 | 163,1 | 4876 | 1707,66 | 476,4 | 617,2 | 13676 | 4923,32 | 309,2 | 291,0 | 3923,9 |
| Assulina muscorum | ASSMUS | 714,6 | 167,3 | 1947 | 943,019 | 223 | 693,9 | 385,3 | 434,094 | 937,6 | 861,2 | 2332 | 1377,11 | 491,6 | -526,6 | 1561,8 |
| Euglypha compressa | EUGCOM | 374,6 | 313,5 | 336,8 | 341,626 | 185,9 | 91,72 | 501,8 | 259,808 | 560,6 | 405,2 | 838,5 | 601,434 | 188,7 | 221,8 | -165,0 |
| Nebela militaris | NEBMIL | 240,4 | 304 | 212,7 | 252,339 | 167,1 | 78,68 | 346,2 | 197,322 | 407,5 | 382,6 | 558,9 | 449,662 | 73,3 | 225,3 | -133,5 |
| Hyalosphenia elegans | HYAELE | 1398 | 151,2 | 238,9 | 595,904 | 177 | 64,27 | 54,8 | 98,6978 | 1575 | 215,4 | 293,7 | 694,601 | 1220,6 | 86,9 | 184,1 |
| Euglypha tuberculata | EUGTUB | 307,2 | 62,37 | 2794 | 1054,37 | 87,41 | 35,97 | 973 | 365,449 | 394,6 | 98,34 | 3767 | 1419,82 | 219,7 | 26,4 | 1820,6 |
| Euglypha ciliata | EUGCIL | 169,1 | 47,87 | 25,37 | 80,7839 | 165,6 | 220,6 | 11,04 | 132,422 | 334,7 | 268,5 | 36,41 | 213,206 | 3,5 | -172,8 | 14,3 |
| Euglypha strigosa | EUGSTR | 86,24 | 0 | 67,01 | 51,0822 | 63,76 | 66,57 | 89,05 | 73,1246 | 150 | 66,57 | 156,1 | 124,207 | 22,5 | -66,6 | -22,0 |
| Arcella discoides | ARCDIS | 304 | 36,69 | 209,5 | 183,403 | 99,72 | 0 | 253,7 | 117,798 | 403,7 | 36,69 | 463,2 | 301,2 | 204,2 | 36,7 | -44,1 |
| Heleopera sphagni | HELSPH | 142,7 | 19,85 | 31,14 | 64,5571 | 79,22 | 15,23 | 309,7 | 134,707 | 221,9 | 35,09 | 340,8 | 199,264 | 63,5 | 4,6 | -278,5 |
| Euglypha laevis | EUGLAE | 11,28 | 50,78 | 273,5 | 111,865 | 7,132 | 26,09 | 443,4 | 158,86 | 18,42 | 76,87 | 716,9 | 270,725 | 4,2 | 24,7 | -169,8 |
| Assulina scandinavica | ASSSCA | 63,47 | 49,89 | 159,3 | 90,8814 | 29,98 | 23,02 | 204,5 | 85,8165 | 93,44 | 72,91 | 363,7 | 176,698 | 33,5 | 26,9 | -45,2 |
| Euglypha rotunda | EUGROT | 62,22 | 45,77 | 28,16 | 45,3854 | 60,57 | 35,73 | 90,06 | 62,1196 | 122,8 | 81,5 | 118,2 | 107,505 | 1,7 | 10,0 | -61,9 |
| Centropyxis aculeata | CENACU | 100,2 | 52,72 | 0 | 50,9601 | 59,04 | 77,97 | 27,4 | 54,8039 | 159,2 | 130,7 | 27,4 | 105,764 | 41,1 | -25,2 | -27,4 |
| Nebela tincta major | NEBTINMAJ | 0 | 0 | 0 | 0 | 115,8 | 30,35 | 0 | 48,7169 | 115,8 | 30,35 | 0 | 48,7169 | -115,8 | -30,4 | 0,0 |
| Nebela bohemica | NEBBOH | 87,44 | 0 | 0 | 29,1454 | 77,17 | 20,65 | 0 | 32,6074 | 164,6 | 20,65 | 0 | 61,7528 | 10,3 | -20,7 | 0,0 |
| Corythion pulchellum | CORPUL | 19,12 | 5,229 | 521,8 | 182,059 | 4,075 | 10,49 | 18,7 | 11,086 | 23,19 | 15,72 | 540,5 | 193,145 | 15,0 | -5,3 | 503,1 |
| Trinema enchelys | TRIENC | 19,93 | 73,74 | 187,6 | 93,7709 | 15,36 | 10,55 | 88,9 | 38,2691 | 35,29 | 84,29 | 276,5 | 132,04 | 4,6 | 63,2 | 98,7 |
| Physochila griseola | PHYGRI | 72,97 | 37,57 | 165 | 91,8628 | 22,63 | 8,241 | 37,86 | 22,9093 | 95,6 | 45,82 | 202,9 | 114,772 | 50,3 | 29,3 | 127,2 |
| Heleopera rosea | HELROS | 30,42 | 47,32 | 12,36 | 30,0329 | 8,013 | 5,668 | 25,95 | 13,2108 | 38,43 | 52,99 | 38,31 | 43,2437 | 22,4 | 41,7 | -13,6 |
| Euglypha cristata | EUGCRI | 64,09 | 6,332 | 126,4 | 65,6204 | 13,29 | 4,764 | 71,83 | 29,9605 | 77,37 | 11,1 | 198,3 | 95,5809 | 50,8 | 1,6 | 54,6 |
| Cyclopyxis arcelloides | CYCARC | 0 | 0 | 44,79 | 14,9284 | 34,87 | 3,373 | 0 | 12,7487 | 34,87 | 3,373 | 44,79 | 27,6771 | -34,9 | -3,4 | 44,8 |
| Phryganella acropodia | PHRACR | 0 | 0 | 0 | 0 | 0 | 56,34 | 0 | 18,7812 | 0 | 56,34 | 0 | 18,7812 | 0,0 | -56,3 | 0,0 |
| Bullinularia indica | BULIND | 0 | 0 | 0 | 0 | 19,64 | 2,14 | 0 | 7,25943 | 19,64 | 2,14 | 0 | 7,25943 | -19,6 | -2,1 | 0,0 |
| Nebela flabellulum | NEBFLA | 0 | 3,05 | 0 | 1,01654 | 3,41 | 9,155 | 25,48 | 12,6806 | 3,41 | 12,2 | 25,48 | 13,6971 | -3,4 | -6,1 | -25,5 |
| Sphenoderia fissirostris | SPHFIS | 0 | 11,68 | 8,457 | 6,71276 | 3,566 | 0 | 0 | 1,18873 | 3,566 | 11,68 | 8,457 | 7,9015 | -3,6 | 11,7 | 8,5 |
| Euglypha sp. | EUGSP | 7,928 | 0 | 0 | 2,64276 | 0 | 7,79 | 0 | 2,59677 | 7,928 | 7,79 | 0 | 5,23953 | 7,9 | -7,8 | 0,0 |
| Trigonopyxis arcula | TRIARC | 3,964 | 0 | 43,28 | 15,7476 | 11,82 | 0 | 0 | 3,94119 | 15,79 | 0 | 43,28 | 19,6888 | -7,9 | 0,0 | 43,3 |
| Heleopera sylvatica | HELSYL | 3,964 | 0 | 0 | 1,32138 | 2,122 | 0 | 0 | 0,70717 | 6,086 | 0 | 0 | 2,02855 | 1,8 | 0,0 | 0,0 |
| Hyalosphenia subflava | HYASUB | 0 | 8,434 | 43,28 | 17,2377 | 1,321 | 3,531 | 0 | 1,61762 | 1,321 | 11,97 | 43,28 | 18,8553 | -1,3 | 4,9 | 43,3 |
| Arcella catinus | ARCCAT | 2467 | 1,703 | 0 | 822,888 | 1306 | 0 | 0 | 435,4 | 3773 | 1,703 | 0 | 1258,29 | 1160,8 | 1,7 | 0,0 |
| Cryptodifflugia oviformis | CRYOVI | 3,269 | 1,151 | 0 | 1,47339 | 0 | 0 | 0 | 0 | 3,269 | 1,151 | 0 | 1,47339 | 3,3 | 1,2 | 0,0 |
| Arcella vulgaris | ARCVUL | 8,718 | 0 | 0 | 2,90585 | 0 | 0 | 0 | 0 | 8,718 | 0 | 0 | 2,90585 | 8,7 | 0,0 | 0,0 |
| Difflugia pulex | DIFPUX | 0 | 0 | 0 | 0 | 0 | 2,43 | 0 | 0,80994 | 0 | 2,43 | 0 | 0,80994 | 0,0 | -2,4 | 0,0 |
| Centropyxis aerophila | CENAER | 0 | 0 | 50 | 16,6651 | 0 | 0 | 0 | 0 | 0 | 0 | 50 | 16,6651 | 0,0 | 0,0 | 50,0 |
| Nebela collaris | NEBCOL | 0 | 0 | 242,7 | 80,9015 | 0 | 0 | 0 | 0 | 0 | 0 | 242,7 | 80,9015 | 0,0 | 0,0 | 242,7 |
| The following taxa are only recorded as dead (empty) shells: Arcella hemispherica, A. rotunda, Cyclopyxis eurystoma, Difflugia lanceolata, D. elegans, D. globulosa, Heleopera petricola, Padaungiella tubulata (=Nebela tubulata), Phryganella acropodia, Pseudodifflugia gracilis, Trinema lineare. | | | | | | | | | | | | | | | | |
