# Supplementary material for: Response of Sphagnum Peatland Testate Amoebae to a 1-Year Transplantation Experiment Along an Artificial Hydrological Gradient
Source: Microb Ecol. 2014 Feb 1;67(4):810–8. doi: 10.1007/s00248-014-0367-8 (PMC3984440; doi:10.1007/s00248-014-0367-8)
Supplement: Supplementary file 4 — Summary results of the RDA model on testate amoeba community data showing changes in significance of the origin (hummock, lawn and pool), seeding (adding pooled community extract from hummock, lawn and pool) and local condition (depth to water table measured in the experimental plots) over the course of the experiment (T0, August 2008; T1, May 2009 and T2, August 2009). Significant values are indicated in bold. (DOCX 11.9 kb) [file 248_2014_367_MOESM4_ESM.docx]

| **Table 1.** Summary results of the RDA model on testate amoeba community data showing changes in significance of the origin (hummock, lawn, pool), seeding (adding pooled community extract from hummock, lawn & pool) and local condition (depth to water table measured in the experimental plots) over the course of the experiment (T0 : August 2008, T1: May 2009 and T2: August 2009). Significant values are indicated in bold. | | | | | | |
| --- | --- | --- | --- | --- | --- | --- |
|  |  |  | P-values | | | |
| Time | R2 | R2-adj | Model | Origin | Seeding | DWT * |
| T0 | 0,130 | 0,113 | **0,005** | **0,001** | **0,017** | 0,326 |
| T1 | 0,142 | 0,125 | **0,005** | **0,001** | **0,003** | 0,655 |
| T2 | 0,101 | 0,084 | **0,005** | **0,001** | 0,474 | **0,002** |
| * Measured DWT (quantitative variable) | | | | |  |  |
