# Supplementary figures and images for: Response of Sphagnum Peatland Testate Amoebae to a 1-Year Transplantation Experiment Along an Artificial Hydrological Gradient
Source: Microb Ecol. 2014 Feb 1;67(4):810–8. doi: 10.1007/s00248-014-0367-8 (PMC3984440; doi:10.1007/s00248-014-0367-8)

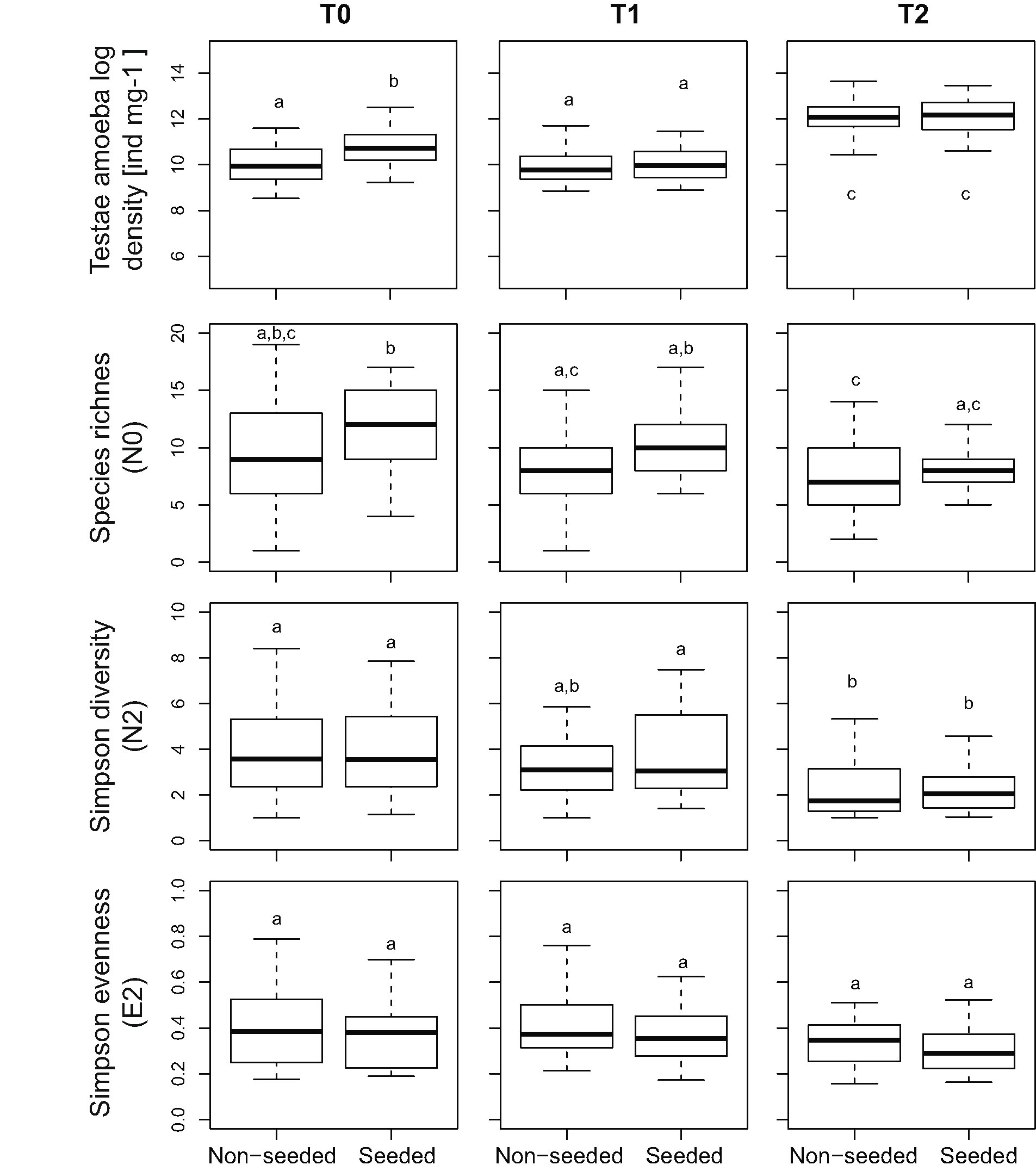

Supplement: Supplementary file 5 — Changes over time in density [ind/mg], species richness (N0), Simpson diversity (N2) and evenness (E2 = N2/N0) of living testate amoebae of communities seeded vs. non-seeded with mixed extract from hummock, lawn and pool habitats, showing data of samples from hummock, lawn and pool habitats (origin) placed at high, intermediate and low water table position (local condition) in the experimental trenches of Le Russey bog, French Jura. T0, August 2008; T1, May 2009 and T2, August 2009. (JPEG 378 kb) [file 248_2014_367_Fig5_ESM.jpg]

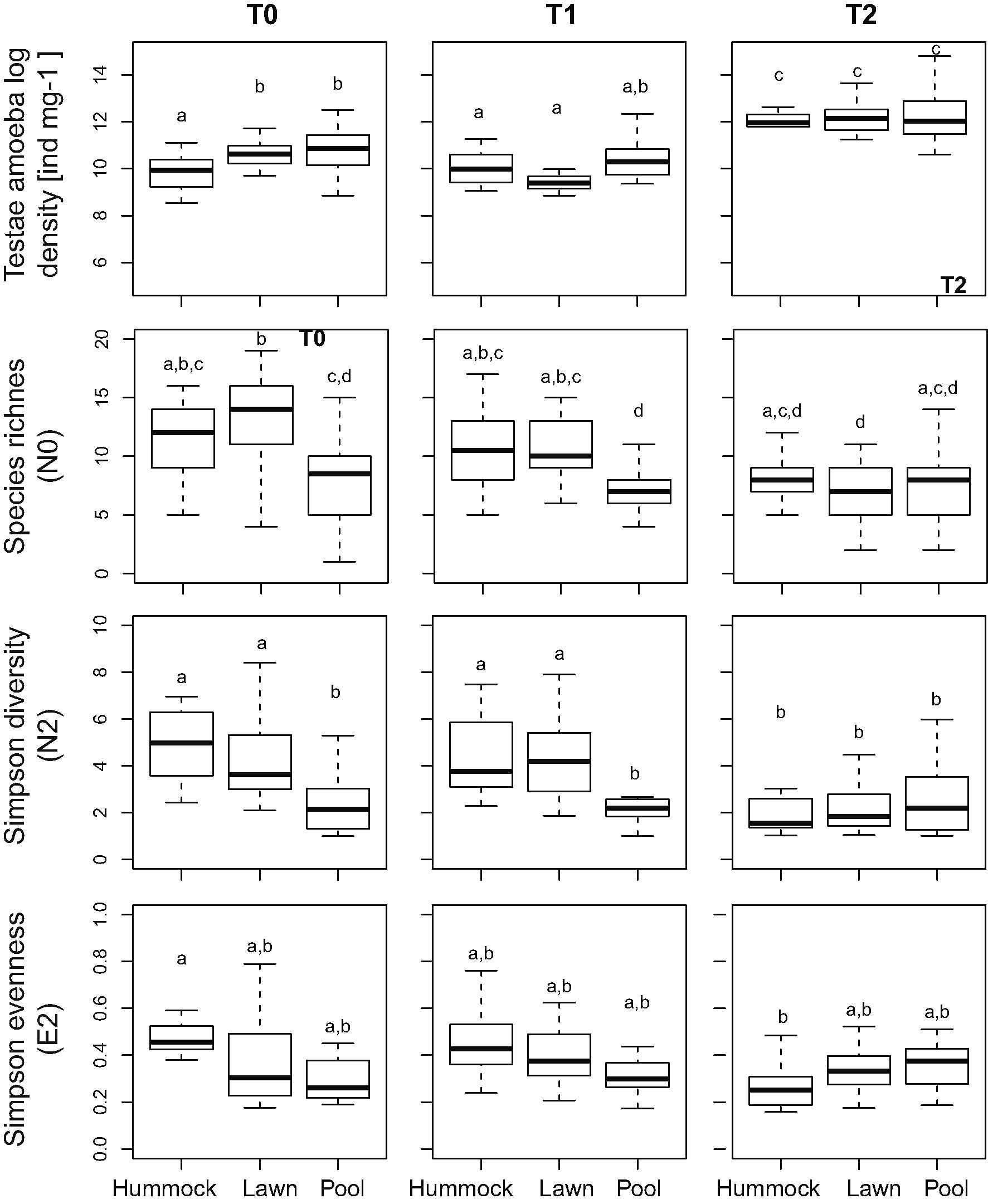

Supplement: Supplementary file 7 — Changes over time in density [ind/mg], species richness (N0), Simpson diversity (N2) and evenness (E2 = N2/N0) of living testate amoebae for samples collected in hummock, lawn and pool habitats, showing data of samples seeded and not seeded with mixed extract from the three habitats (origin) and placed at high, intermediate and low water table position (local condition) in the experimental trenches of Le Russey bog, French Jura. T0, August 2008; T1, May 2009 and T2, August 2009. (JPEG 397 kb) [file 248_2014_367_Fig6_ESM.jpg]

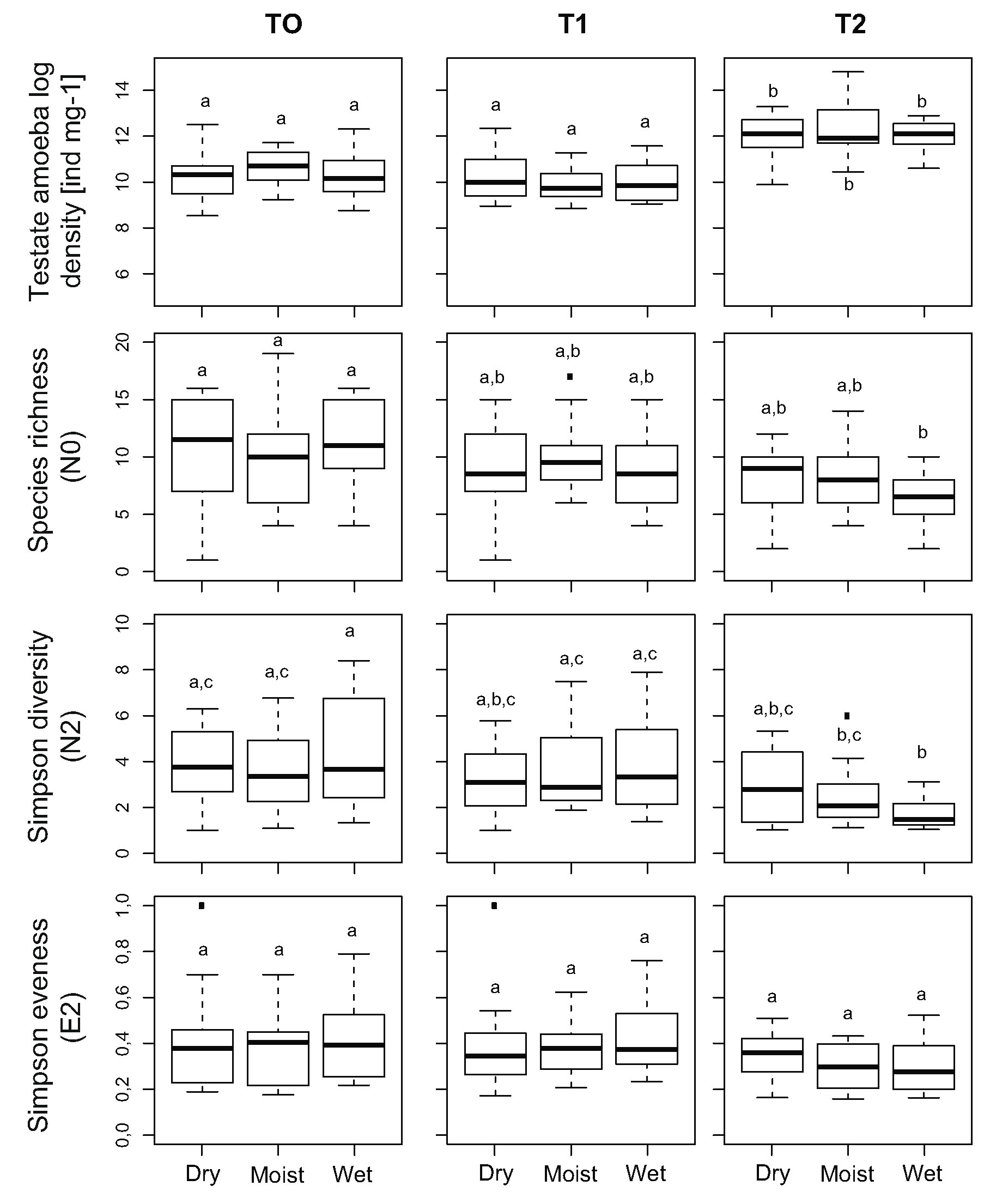

Supplement: Supplementary file 9 — Changes over time in density [ind/mg], species richness (N0), Simpson diversity (N2) and evenness (E2 = N2/N0) of living testate amoebae for samples placed at high, intermediate and low water table position (local condition) the experimental trenches of Le Russey bog, French Jura, showing data of samples collected in hummock, lawn and pool habitats (origin) seeded and not seeded with mixed extract from the three habitats. T0, August 2008; T1, May 2009 and T2, August 2009. (JPEG 372 kb) [file 248_2014_367_Fig7_ESM.jpg]

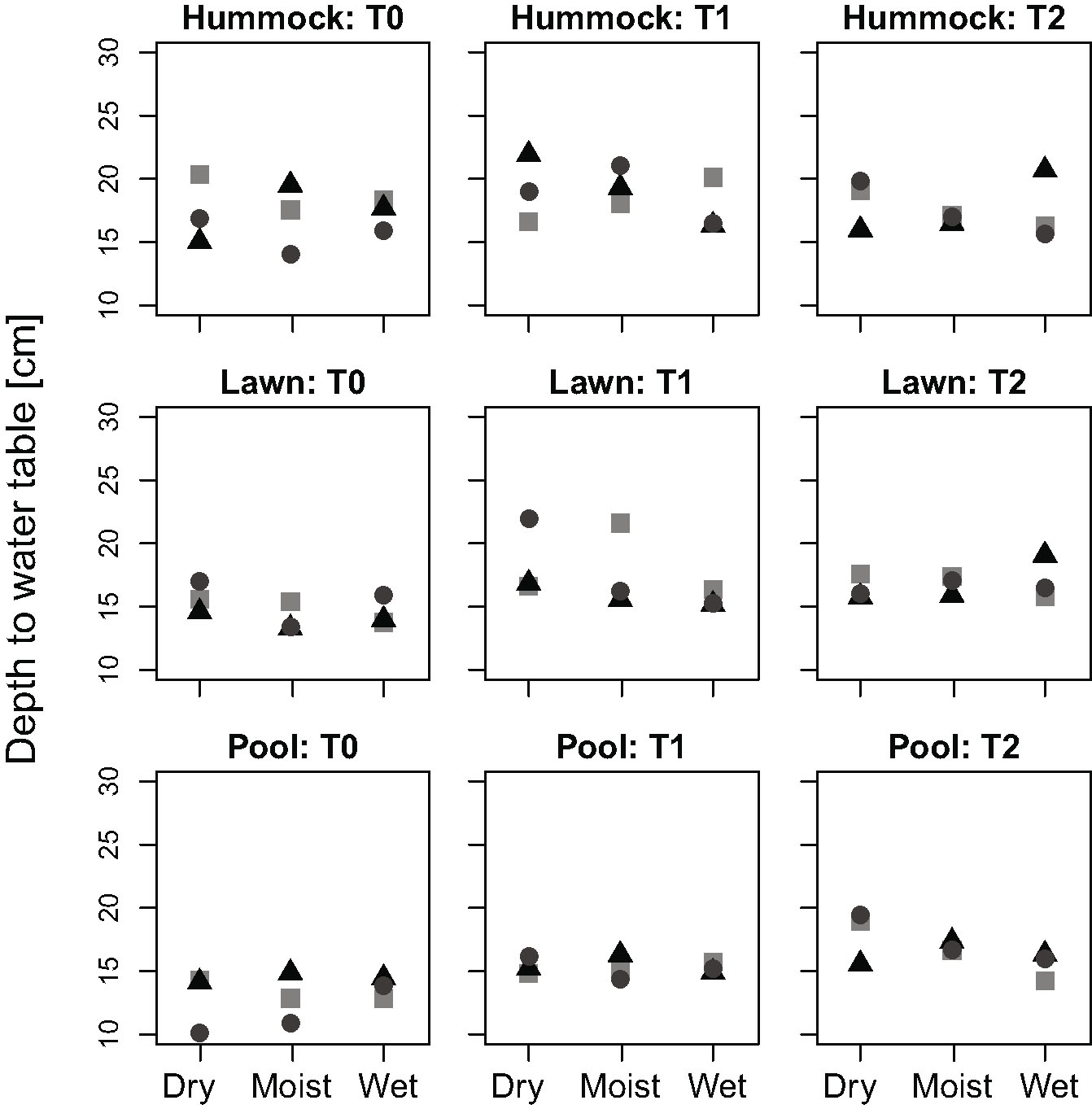

Supplement: Supplementary file 11 — Depth to water table (DWT, in cm) inferred (using the transfer function model from Engadine [40]), from testate amoeba communities sampled at T0, T1 and T2 in the plots of different origins (hummock, lawn and pool) placed at different position (D dry, M moist, W wet). Data from communities seeded with mixed extract from hummock, lawn and pool habitats. See Fig. 4 for corresponding figure on non-seeded communities. (JPEG 196 kb) [file 248_2014_367_Fig8_ESM.jpg]
